# Novel ^68^Ga-FAPI PET/CT Offers Oncologic Staging Without COVID-19 Vaccine–Related Pitfalls

**DOI:** 10.2967/jnumed.122.264872

**Published:** 2023-03

**Authors:** Tristan T. Demmert, Ines Maric, Kelsey L. Pomykala, Katharina Lueckerath, Jens Siveke, Benedikt M. Schaarschmidt, Rainer Hamacher, Ken Herrmann, Wolfgang P. Fendler

**Affiliations:** 1 Department of Nuclear Medicine, West German Cancer Center, University of Duisburg–Essen, Essen, Germany;; 2 German Cancer Consortium, Partner Site Essen University Hospital, and German Cancer Research Center, Essen, Germany;; 3 Institute for AI in Medicine, University Medicine Essen, Essen, Germany;; 4 Bridge Institute of Experimental Tumor Therapy, West German Cancer Center, Essen University Hospital, Essen, Germany;; 5 Department of Diagnostic and Interventional Radiology and Neuroradiology, Essen University Hospital, University of Duisburg–Essen, Essen, Germany; and; 6 Department of Medical Oncology, West German Cancer Center, Essen University Hospital, Essen, Germany

**Keywords:** PET, PET/CT, COVID-19, tumor staging, vaccine-related pitfalls

## Abstract

In the setting of ongoing coronavirus disease 2019 vaccination, vaccine-related tracer uptake in locoregional lymph nodes has become a well-known issue in tumor staging by ^18^F-FDG PET/CT. ^68^Ga-fibroblast-activation protein inhibitor (FAPI) PET/CT is a new oncologic imaging tool that may overcome this limitation. **Methods:** We assessed postvaccine head-to-head and same-day ^18^F-FDG and ^68^Ga-FAPI-46 PET/CT findings in a series of 11 patients from a large, prospective imaging registry. All patients with documented tracer uptake in locoregional lymph nodes on PET/CT or PET/MRI, after vaccination within 6 wk, were eligible for investigation. **Result:** Significant visual lymph node uptake adjacent to the injection site was noted in 11 of 11 (100%) patients with ^18^F-FDG PET/CT, versus 0 of 11 (0%) with ^68^Ga-FAPI PET/CT. ^18^F-FDG detected 73% and ^68^Ga-FAPI PET/CT 94% of all tumor lesions. **Conclusion:** In this case-series study, ^68^Ga-FAPI showed its potential to avoid ^18^F-FDG PET/CT postvaccination pitfalls and presented superior tumor localization.

## Visual Abstract

**Figure:**
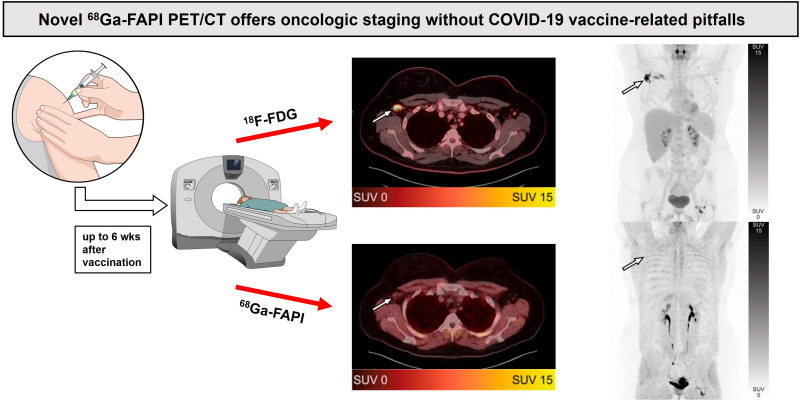

Since the outbreak of the coronavirus disease 2019 (COVID-19) pandemic in 2019 and the start of global mass vaccination in November 2020, several clinical studies have addressed the issue of reactive tracer accumulation in locoregional lymph nodes and upper-arm muscles (1). At local sites, ^18^F-FDG is taken up by immune cells responding to the messenger RNA inflammatory stimulus (2–4). This observation is concerning because vulnerable groups, such as oncologic patients, undergo both regular booster shots and medical imaging. False-positive findings on ^18^F-FDG PET due to uptake in inflammatory cells may trigger false management decisions.

^68^Ga-fibroblast-activation protein inhibitor (FAPI) PET/CT is a novel imaging test directed at cancer-associated fibroblasts in the tumor stroma. Because of its unique mechanism targeting only activated fibroblasts and subtypes of cancer-associated fibroblasts, ^68^Ga-FAPI PET/CT may be able to avoid false-positive postvaccine uptake. ^68^Ga-FAPI PET/CT has emerged as a potential alternative to ^18^F-FDG PET/CT in many tumor types and may avoid locoregional pitfalls caused by vaccination. Here, we assess same-day head-to-head postvaccine ^18^F-FDG and ^68^Ga-FAPI PET/CT uptake in patients from a large, prospective registry of oncologic imaging collected during the ongoing mass vaccination campaign in Germany.

## MATERIALS AND METHODS

**FIGURE 1.**
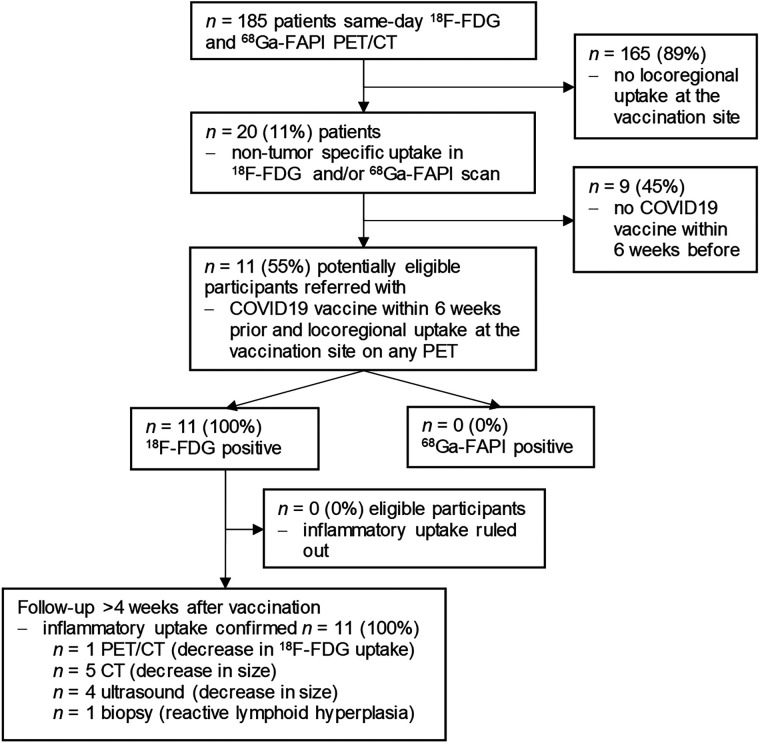
Patient flowchart.

We selected 11 patients with ^68^Ga-FAPI and ^18^F-FDG PET/CT (May 2021 to April 2022) from our prospective database, which is part of a large, prospective observational study (NCT04571086). Enrollment is offered to all patients who undergo ^68^Ga-FAPI-46 PET in our department. Eleven patients met the following criteria: same-day ^68^Ga-FAPI and ^18^F-FDG PET for oncologic staging or restaging, with ^18^F-FDG at least 4 h after ^68^Ga-FAPI PET; ^68^Ga-FAPI or ^18^F-FDG tracer uptake in local soft tissue or nodes, with a visually positive target-to-background ratio on PET/CT; COVID-19 vaccination within 6 wk; and no change in treatment between PET and vaccination (Fig. 1).

The study was approved by the ethics committee (approvals 20-9485-BO and 19-8991-BO), and all patients gave written informed consent for enrollment into a prospective observational trial (NCT04571086).

Lymph nodes were considered positive when demonstrating visually focal uptake above the background level. Visual readings were performed by 2 nuclear medicine physicians in consensus. The median injected dose was 333 MBq (interquartile range [IQR], 245–421 MBq) for ^18^F-FDG and 102 MBq (IQR, 79–125 MBq) for ^68^Ga-FAPI. Follow-up data included imaging and clinical information. All patients fasted for 6 h before the ^18^F-FDG scan, and blood glucose level (<200 mg/dL) was measured. Descriptive statistics are provided. An ANOVA was applied for assessing differences in SUV among the different time frames after vaccination. The SUV was measured in the center of each lymph node, determined on PET/CT images.

Six (55%) patients underwent imaging on a Siemens Biograph Vision and 5 (45%) on a Siemens Biograph mCT device. SUV_peak_ was selected on the basis of phantom cross-calibration for the PET/CT devices to achieve reproducible results.

## RESULTS

Patient characteristics are shown in Supplemental Table 1 (supplemental materials are available at http://jnm.snmjournals.org). After database screening, 11 patients (5 men and 6 women) were included. The mean age was 44 y (IQR, 34–54 y). Three (27%) underwent PET for staging and 8 (73%) for restaging. Five (45%) patients had sarcoma, and 6 (55%) patients had carcinoma. When combined findings from ^68^Ga-FAPI and ^18^F-FDG scans were considered, 3 (27%) patients showed primary lesions only, 1 (9%) patient showed locoregional lesions, and 7 (64%) patients showed distant metastases. Distant metastatic disease was noted in visceral organs, bone, and soft tissue in 5 (45%), 1 (9%), and 1 (9%) patients, respectively. The thoracic cavity was tumor-free in 7 (64%) patients. Before chemotherapy, 2 (18%) of 11 patients had a thoracic primary tumor, that is, lung (*n* = 1) and breast cancer (*n* = 1), which have a higher likelihood of axillary nodal involvement (Supplemental Table 2).

**FIGURE 2.**
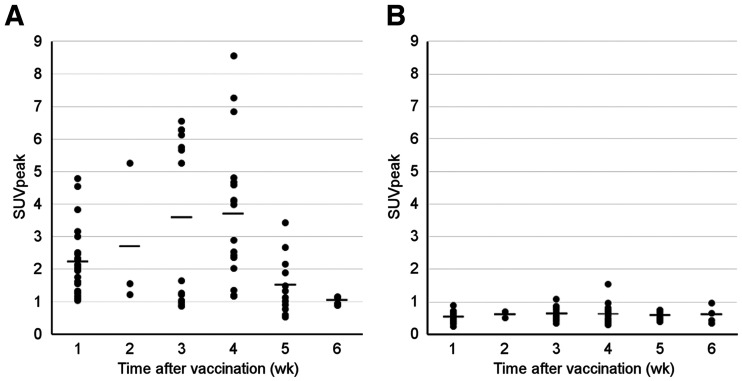
SUV_peak_ over time for 78 locoregional lymph nodes for ^18^F-FDG (ANOVA *P* = 0.002) (A) and ^68^Ga-FAPI (ANOVA *P* = 0.791) (B) PET/CT. Average SUV_peak_ for weeks 1, 2, 3, 4, 5, and 6 was 2.2, 2.7, 3.6, 3.7, 1.5, and 1.0, respectively, for ^18^F-FDG PET and 0.5, 0.6, 0.6, 0.6, 0.6, and 0.6, respectively, for ^68^Ga-FAPI PET.

Ten (91%) patients received BNT162b2 vaccine and 1 (9%) received messenger RNA1273 vaccine at a median interval of 19 d (IQR, 8–30 d) before PET/CT. Eleven (100%) patients demonstrated focal ^18^F-FDG tracer uptake in axillary lymph nodes on PET/CT; none of the patients had focal ^68^Ga-FAPI uptake. Details are listed in Supplemental Table 3. SUV at different times after vaccination (Fig. 2A) demonstrated the highest ^18^F-FDG accumulation in lymph nodes at 2–4 wk after vaccination (ANOVA *P* = 0.002), whereas no increase in uptake on ^68^Ga-FAPI was observed at any of the time points (Fig. 2B, ANOVA *P* = 0.79).

Imaging follow-up data at an average of 120 d (IQR, 44–196 d) confirmed reactive nodal uptake in all patients: a decrease in uptake was documented in all 4 (100%) lymph nodes of the patient who underwent follow-up ^18^F-FDG PET/CT. A decrease in lymph node size was documented for all patients (on CT, *n* = 5 [100%]; on ultrasound, *n* = 4 [100%]). One patient underwent a biopsy confirming reactive lymphoid hyperplasia with no evidence of malignancy (Supplemental Fig. 1). Further tracer accumulation at the injection site in the deltoid muscle was detected in 5 (45%) patients (Supplemental Fig. 2.2). Another patient showed generalized tracer accumulation in the bone marrow on ^18^F-FDG PET in addition to splenic uptake above the level of liver uptake, indicating reactive bone marrow and splenic activation by a vaccine-induced immune response (Supplemental Fig. 3). According to combined ^18^F-FDG and ^68^Ga-FAPI PET/CT reports, none of the patients had tumor involvement of the arm or axillary lymph nodes. One patient with breast cancer demonstrated new bone metastases 4 y after initial therapy. Local recurrence was not noted, and focal nodal uptake was seen ipsilateral to the vaccination site and contralateral to the former tumor site.

The combined analysis of ^68^Ga-FAPI and ^18^F-FDG scans detected, in total, 102 (100%) tumor lesions (primary, 6 [6%]; locoregional, 26 [25%]; distant nodal, 10 [10%]; lung, 7 [7%]; liver, 18 [18%]; bone, 28 [27%]; and soft tissue, 7 [7%]). Lesion detection efficacy was higher for ^68^Ga-FAPI than for ^18^F-FDG PET (96 [94%] vs. 74 [73%]). ^68^Ga-FAPI PET detected additional tumor lesions in the lung (7 [100%] vs. 5 [71%]), liver (17 [94%] vs. 9 [50%]), and bone (28 [100%] vs. 23 [82%]). The superior efficacy was based on a higher detection rate in 3 patients with different tumor entities (ovarian cancer, solitary fibrous tumor, and breast cancer). There was no ^18^F-FDG–positive, ^68^Ga-FAPI–negative primary tumor lesion (Supplemental Table 4).

Representative images are shown for all included patients in Supplemental Figures 1–11.

## DISCUSSION

The COVID-19 pandemic is active globally, with an estimated 12.8 billion vaccine doses given and 627.1 million registered infections as of October 21, 2022 (5). Vaccines aim to decrease COVID-19 spread and severe disease, protecting vulnerable groups, including cancer patients (6). Repeat vaccinations have been endorsed by the Centers for Disease Control and many national infection agencies (7). Thus, most cancer patients underwent 3 vaccinations within the last year (7). On the basis of current knowledge about decreasing protection over time and the emergence of new variants, vaccination at least annually is the likely scenario (8–10). For vulnerable groups, repeat COVID-19 vaccination will come in addition to annual flu shots.

Postvaccination lymph node uptake on ^18^F-FDG PET has been demonstrated in prior case reports or case series on COVID-19 and flu vaccine for a variety of tumor entities (1–4,11–16). An increase in annual vaccination will put oncologic patients at considerable risk of false-positive ^18^F-FDG PET findings. Here, we confirmed ^18^F-FDG uptake within 6 wk of vaccination in a case series of oncologic patients. Nonspecific ^18^F-FDG uptake will adversely influence staging and restaging procedures in oncology patients (3). Patients are at risk of false-positive lymph node findings when undergoing ^18^F-FDG PET/CT within 6 wk of vaccination. Therefore, PET/CT appointments for patients at risk must be planned carefully with consideration of any vaccination. In addition, bone marrow uptake was seen in 1 patient, pointing to an additional pitfall in patients with myeloproliferative disease (2).

The regulation of fibroblast activation protein α in cancer is not completely understood. Transforming growth factor β, associated with epithelial–mesenchymal transition, angiogenesis, or immune suppression, participates in the upregulation of fibroblast activation protein α expression, suggesting a lower susceptibility of ^68^Ga-FAPI PET to acute inflammation (17). Fittingly, none of the patients demonstrated focal ^68^Ga-FAPI uptake in locoregional lymph nodes after vaccination. In addition, ^68^Ga-FAPI PET demonstrated higher detection efficacy when compared with ^18^F-FDG PET/CT both for locoregional and for distant staging. Tumor detection was based on the PET/CT findings; however, lesions were not verified by imaging follow-up, and findings are limited by a low sample size. Superior detection for ^68^Ga-FAPI versus ^18^F-FDG PET is in line with previous reports on carcinoma of unknown primary, sarcoma, and breast carcinoma imaging (18). Our findings indicate that ^68^Ga-FAPI PET delivers oncologic staging with accuracy equal or superior to that of ^18^F-FDG PET but with no risk of a false diagnosis after vaccination (19).

An ongoing prospective trial at our institution aims to assess accuracy and correlation with histopathology for various types of cancer (clinicaltrials.gov, NCT05160051). Our study was limited by a low number of patients and a low histopathologic confirmation rate for lymph node findings.

## CONCLUSION

Increased annual vaccinations are expected for vulnerable groups, including cancer patients. ^18^F-FDG may trigger costly follow-up investigations and false management decisions. In our study, ^68^Ga-FAPI PET, a promising novel imaging tool, avoided postvaccination lymph node and bone marrow pitfalls and provided accurate oncologic staging. ^68^Ga-FAPI PET should be assessed as an alternative to ^18^F-FDG PET in ongoing (NCT05160051) and future prospective studies.

## DISCLOSURE

Katharina Lueckerath reports fees from SOFIE Bioscience (consultant) and Enlaza Therapeutics (consultant). Rainer Hamacher is supported by the Clinician Scientist Program of the University Medicine Essen Clinician Scientist Academy sponsored by the faculty of medicine and Deutsche Forschungsgemeinschaft (DFG) and has received travel grants from Lilly, Novartis, and PharmaMar, as well as fees from Lilly and PharmaMar. Jens Siveke received honoraria as a consultant or for continuing medical education presentations from AstraZeneca, Bayer, Bristol-Myers Squibb, Eisbach Bio, Immunocore, Novartis, Roche/Genentech, and Servier; his institution receives research funding from Bristol-Myers Squibb, Celgene, Eisbach Bio, and Roche/Genentech; he holds ownership and serves on the board of directors of Pharma15. Benedikt Schaarschmidt received a research grant from PharmaCept for an ongoing investigator-initiated study not related to this paper. Ken Herrmann reports personal fees from Bayer, Sofie Biosciences, SIRTEX, Adacap, Curium, Endocyte, BTG, IPSEN, Siemens Healthineers, GE Healthcare, Amgen, Novartis, ymabs, Aktis Oncology, Theragnostics, and Pharma15; other fees from Sofie Biosciences; nonfinancial support from ABX; and grants from BTG. Wolfgang P. Fendler reports fees from Sofie Biosciences (research funding), Janssen (consultant, speakers’ bureau), Calyx (consultant), Bayer (consultant, speakers’ bureau, research funding), Parexel (image review), and AAA (speakers’ bureau). All disclosures were outside the submitted work. No other potential conflict of interest relevant to this article was reported.

## Notes

KEY POINTS **QUESTION:** Can ^68^Ga-FAPI PET prevent COVID-19 vaccine–related reactive lymph node uptake? **PERTINENT FINDINGS:** We compared ^18^F-FDG and ^68^Ga-FAPI PET/CT acquired on the same day within 6 wk of COVID-19 vaccination in 11 oncology patients. Although ^18^F-FDG was visually positive in 11 patients, ^68^Ga-FAPI had higher tumor detection efficacy and showed no vulnerability to vaccine-related tracer uptake in any patients. Additionally, the ^18^F-FDG uptake intensity was time-dependent on the vaccination interval. ^68^Ga-FAPI was visually negative at all time points. **IMPLICATIONS FOR PATIENT CARE:** ^68^Ga-FAPI avoids vaccine-associated reactive lymph node uptake and is therefore superior to ^18^F-FDG in tumor staging up to 6 wk after COVID-19 vaccination.
